# Potential Connectivity of Coldwater Black Coral Communities in the Northern Gulf of Mexico

**DOI:** 10.1371/journal.pone.0156257

**Published:** 2016-05-24

**Authors:** Yuley Cardona, Dannise V. Ruiz-Ramos, Iliana B. Baums, Annalisa Bracco

**Affiliations:** 1 Georgia Institute of Technology, School of Earth and Atmospheric Sciences, 311 Ferst Dr., Atlanta, GA, 30332, United States of America; 2 Departamento de Geociencias y Medio Ambiente, Universidad Nacional de Colombia, Sede Medellín, Colombia; 3 The Pennsylvania State University, Department of Biology, 208 Mueller Laboratory, University Park, PA, 16802, United States of America; Instituto Español de Oceanografía, SPAIN

## Abstract

The black coral *Leiopathes glaberrima* is a foundation species of deep-sea benthic communities but little is known of the longevity of its larvae and the timing of spawning because it inhabits environments deeper than 50 m that are logistically challenging to observe. Here, the potential connectivity of *L*. *glaberrima* in the northern Gulf of Mexico was investigated using a genetic and a physical dispersal model. The genetic analysis focused on data collected at four sites distributed to the east and west of Mississippi Canyon, provided information integrated over many (~10,000) generations and revealed low but detectable realized connectivity. The physical dispersal model simulated the circulation in the northern Gulf at a 1km horizontal resolution with transport-tracking capabilities; virtual larvae were deployed 12 times over the course of 3 years and followed over intervals of 40 days. Connectivity between sites to the east and west of the canyon was hampered by the complex bathymetry, by differences in mean circulation to the east and west of the Mississippi Canyon, and by flow instabilities at scales of a few kilometers. Further, the interannual variability of the flow field surpassed seasonal changes. Together, these results suggest that a) dispersal among sites is limited, b) any recovery in the event of a large perturbation will depend on local larvae produced by surviving individuals, and c) a competency period longer than a month is required for the simulated potential connectivity to match the connectivity from multi-locus genetic data under the hypothesis that connectivity has not changed significantly over the past 10,000 generations.

## Introduction

The management and conservation of deepwater (deeper than 50 m) coral communities requires understanding of how populations are connected [1–8] in environments that are challenging to monitor. Connectivity influences gene flow among coral colonies, their evolution and genetic diversity, controls the probability of speciation, and contributes to their ability to adapt to natural and anthropogenic stresses [9]. For example, in the event of a localized perturbation well-connected populations can deliver larval recruits to disturbed communities and thus aid in their recovery. However, the scale over deepwater coral populations exchange larvae has proven difficult to predict [10–12], complicating our understanding of impact and recovery of those communities from disturbance events [13, 14].

Deepwater corals are mostly restricted to hard substrates upon which they build the structural foundation for many associated species [15] and populations of sessile adult corals are connected only via their planktonic larvae. Corals release larvae into the water column that then disperse and so can connect populations. Their connectivity, therefore, depends on the biological treats of their larvae, and on the dispersal properties of the surrounding environment. Those larvae are generally poor swimmers compared to e.g. fish larvae, and for some deep species have a survival time of about ten days [16], possibly prolonged by a state of low metabolic activity [17, 18]. Those factors taken together challenge the view of large distance dispersal, and therefore cosmopolitan distributions, of deep sea populations [19]. Indeed, many marine taxa show significant population structure, suggesting that connectivity is likely a function of the life history of the species (i.e. timing of spawning, time spent in the water column, settlement behavior), the physical (mean currents, eddies, waves) and biotic (predators, food) environment [20–23].

The northern Gulf of Mexico (GoM) hosts extensive shallow and deepwater coral communities (reviewed in [24–26]). Hard substrate is patchy and ephemeral throughout the deep GoM and therefore connectivity between deepwater communities depends on the larvae ability to cover distances that vary between tens to few hundreds of kilometers. Determining the spatial and temporal scale of influence for a given colony is therefore of primary importance to understand what drives the population dynamics, to inform management decisions to insure the long-term health of local communities, and to predict how those communities might respond to local and remote environmental changes. In the Gulf one example of such disturbances has been the 2010 *Deepwater Horizon* (DWH) oil spill. A wide-range of communities, including deep coral communities (reviewed in [27, 28]) have been impacted by it. Oily material settled on coral branches and killed tissue thus changing the demography of the affected species [29–31]. Consequences of tissue loss likely include reduced reproductive output but the geographic footprint of this reduced output is not known and the footprint will depend in part on the connectivity among populations [28]. However, the scale over which marine invertebrate populations connect in the northern Gulf of Mexico has not been studied. Previous connectivity studies of benthic organisms within the northern GoM suggested panmixia among sites [32–34], but few of them targeted deep sea corals [33].

Numerical hydrodynamic models are powerful tools to explore how larvae released in the water column are dispersed, and to quantify the contribution of physical processes to the potential connectivity among populations (e.g. [35]). Models include uncertainties, they use parameterizations to account for processes at scales that are not directly resolved, and have to be verified against in-situ data. The validation process is usually straightforward near the surface, where satellites continuously map sea surface temperature and sea surface height, in turn linked to geostrophic currents, but can be limited by the availability of quality-controlled in-situ data at depth. In the case of the Gulf of Mexico velocity measurements have been collected through the years through few and sparse in time programs, and more consistently by instruments deployed at oil and gas platforms (e.g. [36]).

In this work we evaluate the dispersal potential of the black coral *Leiopathes glaberrima*, a foundation species of the deep-sea benthos, by comparing results from a numerical model of the ocean circulation that includes a representation of larvae transport with results from a genetic study on realized larvae dispersal. *Leiopathes* colonies are present in the northern Gulf of Mexico in at least four locations named after the Bureau of Ocean Energy Management lease blocks in which they occur: Garden Banks 299 (GB299, 27.69˚N, 92.23˚W), Green Canyon 140 (GC140, 27.81˚N, 91.54˚W), Viosca Knoll 826 (VK826, 29.16˚N, 88.02˚W), and Viosca Knoll 906 (VK906, 29.07˚N, 88.38˚W). Phylogenetic and population genetic analyses of those colonies have shown the presence of two sympatric lineages of *L*. *glaberrima* with restricted realized gene flow [37]. However, the genetic data cannot indicate whether dispersal restriction or post-settlement mortality causes the barriers to gene flow among sites. Here we study the scale over which those sites are potentially connected by simulating larvae dispersal with a high resolution (1 km in the horizontal) regional ocean model. We investigate the role played by the timing of spawning, larvae lifespan, ocean currents and circulation features. A comparison of the biophysical model results and of the genetic estimates of migration calculated with Migrate-n software [38] completes the effort. Migrate-n uses single-locus or multi-loci data to estimate migration rates between populations and estimate all parameters required through maximum likelihood or Bayesian inference.

A better understanding of the geographic footprint of larval dispersal will lead to smarter management decisions and a more in-depth understanding of the processes that structure deep sea ecosystems, and this is especially important in areas susceptible to large disturbance events such as the Gulf of Mexico.

## Material and Methods

Connectivity can be explored in several complementary ways [39]. In this work we evaluate it with a genetic study on realized larval dispersal [37] and using a numerical model of the ocean circulation and larvae transport tracking algorithms [35, 40]. It is important to note that the two methodologies vary widely in the temporal scale addressed: the genetic data integrates successful dispersal events over many (~ 10,000) generations while the hydrodynamic model was run for three recent years.

### Genetic data

Genetic data on 207 samples representing different morphotypes of *Leiopathes glaberrima* are described in detail in [37]. Briefly, *L*. *glaberrima* colonies were collected by the remotely operated vehicle (ROV) *Jason II* from the R/V Ron Brown (August 6 to September 12, 2009 and October 15 to November 1, 2010), and by the manned submersible *Johnson Sea Link* from the R/V Seward Johnson (September 16 to 23, 2009 and September 22 to October 2, 2010). The 207 samples of *L*. *glaberrima* were collected from depths between 248 m and 674 m, from GB299 (N = 27), GC140 (N = 23), VK826 (N = 75), and VK906 (N = 73) (Table 1 in [37]). Tissue was preserved in 70% ethanol and stored at -80°C. Permitting processes are not established for work on deep-sea corals in the Gulf of Mexico. Letters of acknowledgment were obtained from the National Oceanic and Atmospheric Administration for the research expeditions in accordance with the Magnuson-Stevens Fishery Conservation and Management Act.

Coral samples were genotyped using 10 polymorphic microsatellite loci (Genbank submission numbers KJ914618-KJ914627). Fragments were analyzed using an ABI 3730 sequencer with an internal size standard (Genescan LIZ-500; Applied Biosystems). Genemapper 4.0 (Applied Biosystems) was used to visualize the electropherograms and score the alleles. Samples that failed to amplify for more than 2 loci (n = 28, 13%) were excluded from the analyses.

Genetic analyses were performed using the program GenAlEx v6.5 [41]. Exact matches at all loci were identified first and samples that shared the exact multilocus genotype (MLG) at all 10 loci were considered to be clonemates of the same genet. Using all 10 loci, GenAlEx estimated the probability of identity (or the probability that two samples share the same MLG even though they are not clones) as 10^−7^ across populations. Only one MLG from each genet was retained for population genetic analysis (n = 137 MLGs remained). Unsupervised clustering of genotypes was performed in Structure [42]. In this paper, migrate-n was used to estimate the number of migrants among populations [38, 43] using 4 heated chains (starting “temperatures” 1.0, 1.2, 3.0, 10^6^, where ‘temperature’ is a non-dimensional variable used in Metropolis-coupled Markov chain Monte Carlo affecting the likelihood of acceptance of a proposed genealogy tree such that the “hotter” chains exploring more genealogy space than the “cold” chains) and one long chain with 5000 steps and burn-in of 10000. Estimated migration rates were also calculated with BayesAss and results are included in the Supporting Information (S1 Table).

*L*. *glaberrima* is a genetically diverse species consisting of two partially sympatric genetic lineages in the northern GoM that employ different mating strategies [37]. While there is evidence from microsatellite loci that gene flow among the two lineages is restricted, morphological, nuclear and mitochondrial sequence markers point to one species consisting of geographically structured populations. Here, genetic lineages were treated as different subspecies of *L*. *glabberima*, and considered separately in the migration analyses. For comparative purposes, genetic lineages were also considered together.

The genetic data relevant to this work may be obtained through the Gulf of Mexico Research Initiative Information and Data Cooperative (GRIIDC), doi:10.7266/N7QJ7F82.

### Configuration of the ocean circulation model

**Fig 1:**
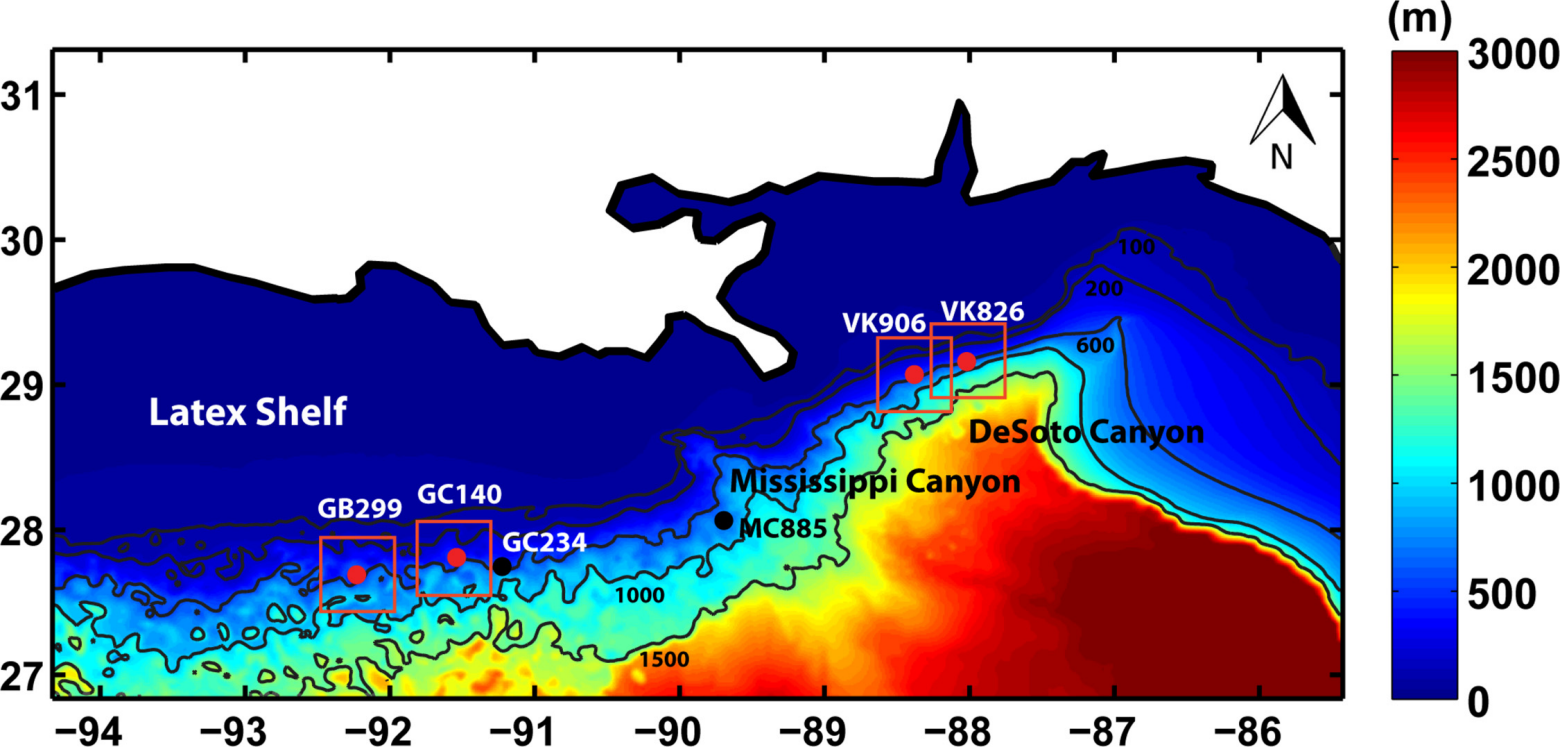
Model domain. ROMS was configured over the region for which the complex bathymetry is shown above. The Louisiana and Texas (LATEX) shelf and major canyons are also indicated. Lagrangian particles were then released within the areas marked by the red squares and allowed to disperse for 40 days.

The Regional Ocean Modeling System (ROMS) was used to advect neutrally buoyant Lagrangian particles (i.e. particles that can freely move in all directions in the water column), referred to as “virtual larvae”, in the northern Gulf of Mexico. ROMS is a 3-dimensional, free-surface, hydrostatic, primitive-equation model with a generalized vertical terrain-following coordinate [44]. The modeled region (Fig 1) extends from 94.33° W to 85.42° W and from 26.84° N to 31.31° N encompassing the Louisiana and Texas shelves (or Latex shelf), the Sigsbee escarpment, the De Soto and Mississippi Canyons and the Mississippi Fan, and includes the four coral sampling locations. The model resolution was set to 40 vertical levels with spacing refined at the surface and near the bottom, and 1 km in the horizontal. The horizontal resolution was chosen to resolve the bathymetry details and to partially resolve the submesoscale (100 m–10 km) dynamics that may take place near the ocean bottom [45, 46]. The domain in Fig 1 was nested into the solution presented in the recent published work by Bracco et al. and Luo et al. [46, 47] that covered the whole Gulf. Such “parent” grid provided the velocity fields and stratification at the open boundaries to the east, west and south over the period January 1^st^, 2010–December 31^st^, 2012 on a 5 km horizontal resolution grid every three days. As in [46, 47] the circulation was forced by six hourly winds and daily heat fluxes from the ECMWF-Interim atmospheric reanalysis [48], and the bathymetry was derived from ETOPO1 [49] and smoothed using a Shapiro filter with r_MAX_ of 0.35 [50]. A non-local, K-profile planetary (KPP) boundary layer scheme [51] was used to parameterize the unresolved vertical subgrid-scale processes. Turbulence at the bottom was damped by quadratic bottom drag with coefficient C_d_ = 0.003. It has been shown that modeled eddies are realistically represented when drag is quadratic over a wide range of friction coefficients [52]. Horizontal harmonic mixing of momentum was applied in the horizontal with mixing coefficient varying from 5 m s^-2^ to 25 m s^-2^ to insure that our results were not sensitive to the specific number chosen. A sensitivity analysis proving that this was indeed the case is presented in the Supporting Information (S1 Fig). The range selection is representative of common values found in the literature for comparable model resolutions (e.g. [53, 54]). In the following results are presented for the higher mixing coefficient case that is potentially more favorable to greater dispersal.

The surface salinity field in the model was nudged to the monthly climatology provided by the World Ocean Atlas 2009 (WOA09, [55]), accounting for the seasonal cycle of the fresh water fluxes associated with the Mississippi-Atchafalaya River System but not their interannual variability. Given our focus on the mean behavior of deepwater corals and their larval transport at depth, the representation of the velocity field is particularly important and our conclusions are not limited by excluding interannual variations in salinity.

This configuration further improved that used in [46, 47], where extensive verification of surface fields, stratification and currents below 1000 m can be found, in one respect. In [46, 47] the GOM circulation was simulated with horizontal resolution of 5 km downscaled to 1.6 km in the northern part of the basin, but the bathymetry was scaled to 5 km everywhere and filtered based on the 5 km grid. In [46] we showed that modeled near bottom velocities below 1000 m had mean values in good agreement with the available observations, but at times underestimated the observed intensity in correspondence of steep topographic features ([56–58]), because those features had been smoothed out. The current investigation aims at quantifying the probability of larvae dispersal over few hundred kilometers and few weeks, and localized, episodic near-bottom current intensification events could facilitate connectivity. For this reason we choose to further augment the resolution and to retain the bathymetry details as much as permitted by the grid spacing. Configuration and forcing files are available through the Gulf of Mexico Research Initiative Information and Data Cooperative (GRIIDC), doi: 10.7266/N7V9860S.

### Modeled circulation, variability and transport

#### Observed circulation

The Gulf of Mexico circulation is to a first approximation that of a two-layer system [59], with the upper layer extending to 1000–1200 m depth and encompassing the coral locations. Its mesoscale variability is dominated by the Loop Current (LC), a warm ocean current that enters the basin through the Yucatan Channel and exits it through the Florida Straits, bringing between 23 and 27 sverdrups (1 sverdrup = 10^6^ m^3^s^−1^) of salty and warm waters from the Caribbean Sea into the GoM [60]. At times, the LC can be found confined to the southern portion of the Gulf, others it extends northward, reaching the Louisiana-Mississippi shelf break [61]. The LC transit in the GoM is characterized by the formation of eddies through instabilities and interaction with the bathymetry [59], and, occasionally, by the detachment of large anticyclonic eddies, known as Loop Current eddies or Rings that extend in depth to 800–1000 m. LC eddies usually loose coherency once they reach the continental shelf on the western boundary.

Near the surface, the circulation is driven by weak southeasterly winds that blow towards the coast between April and August and turn to southerly east of the Mississippi River mouth, and by stronger Northeasterlies from September to March [62, 63]. This results in a predominantly counterclockwise circulation in the upper 500–600 m, with zonal, weak and variable currents.

#### Modeled circulation and validation

The current configuration followed closely that presented in [46, 47], as to be expected given that the simulation in [46, 47] provided the boundary conditions. In those papers we showed that the seasonal cycle and intensity of the surface geostrophic velocities, the statistics associated with the variability and eddy shedding of the Loop Current, the water column stratification and the deep circulation were accurately reproduced. The modeled circulation was validated against satellite data and temperature and salinity profiles from Conductivity-Temperature-Depth (CTD) casts obtained during two research cruises that covered the domain under investigation between August 22 and September 15, 2010 (R/V Oceanus, OC468), and July 3 –July 26, 2011 (R/V Endeavor, EN496) (see Fig A2 in [47]). ROMS, even without data assimilation, provided a reliable representation of the physical properties across the water column. Note, however, that in the upper 300 m modeled and observed profiles of salinity differed, since the interannual variability of the Mississippi river inflow was not included. Further, the modeled and observed evolution of the Loop Current cannot and did not coincide because data was not assimilated; this is due to the chaotic nature of the Loop Current [64].

**Fig 2:**
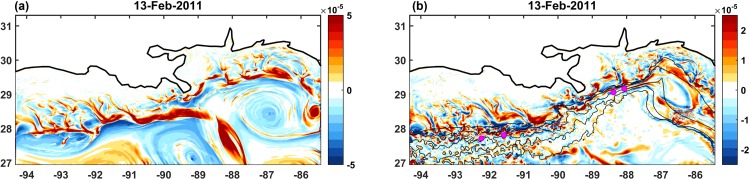
Relative vorticity. (a) Surface and (b) near bottom relative vorticity snapshots in February 2011. Unit: s^-1^. Positive values indicate cyclonic, anticlockwise motion, and negative anticyclonic, clockwise spin. Magenta dots in panel (b) mark the location of coral colonies. Bathymetric isolines as in Fig 1.

Variability generated by mesoscale (scales between 10 and 500 km) and submesoscale perturbations can easily obscure the mean currents at the depths of the coral colonies. Near the ocean surface the mesoscale dynamics influencing our domain are the Loop Current, the large (~ 100 km) anticyclonic Loop Eddies that detach from the Loop Current and can encroach the slope, and smaller (~ 20 km) cyclonic and anticyclonic eddies generated through instabilities of the mean currents interacting with the bathymetry [59]. Their rotational motion can be easily identified in the relative vorticity field, $\omega =\frac{\partial v}{\partial x}-\frac{\partial u}{\partial y}$ where *u* is the zonal (west to east) velocity component and *v* is the meridional (south to north) velocity component, shown in Fig 2. Negative values are representative of anticyclonic or clockwise rotation, and positive values of cyclonic or anticlockwise motion.

Submesoscale turbulence and eddies with a diameter of a few kilometers dominate the vorticity distribution and transport properties along the continental slope (Fig 2B). The variability in their formation and propagation is controlled, mostly, by the periodic intensification of the near-bottom zonal currents over topographic features (e.g. [56–58]).

**Fig 3:**
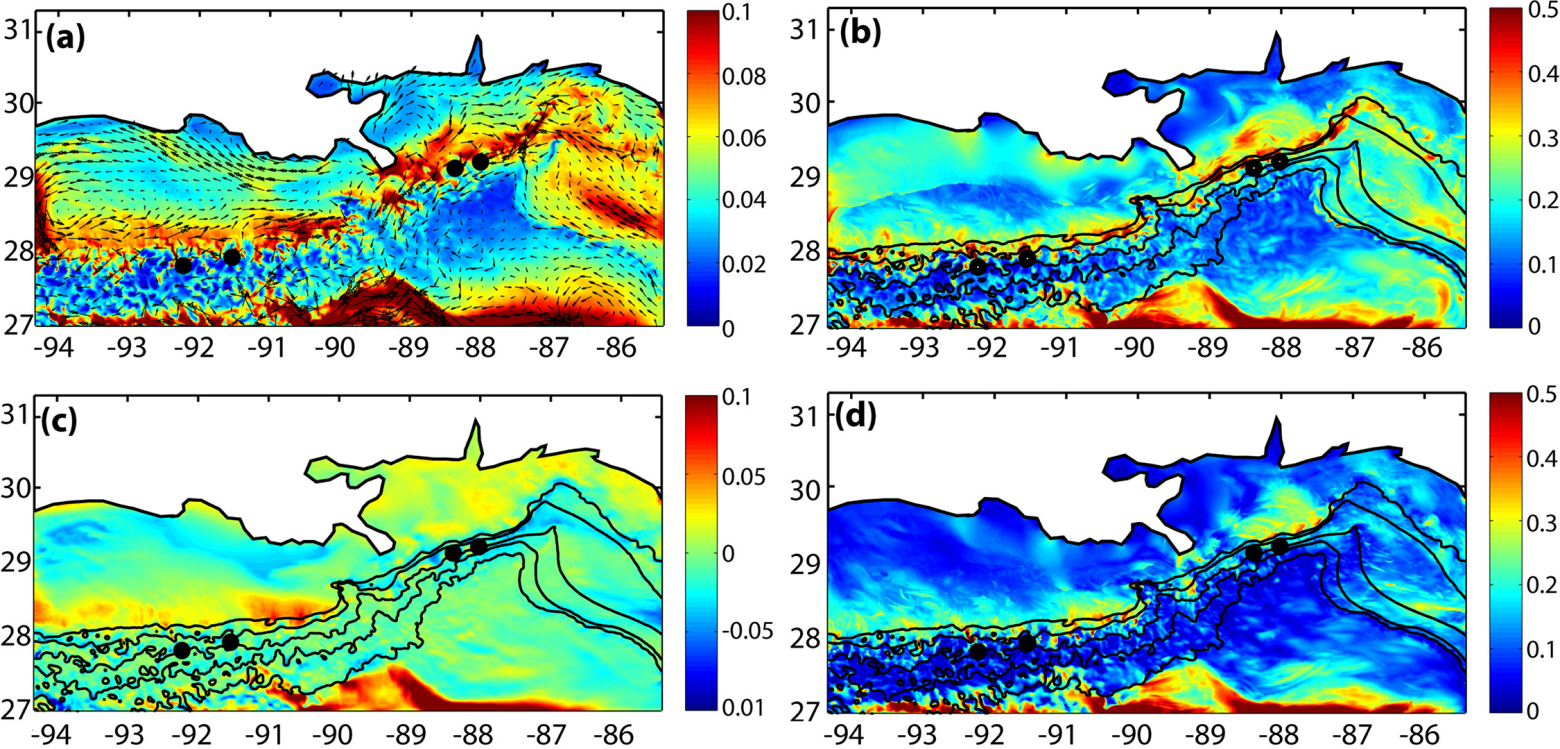
Near bottom speed and zonal velocity. (a) mean speed; (b) maximum speed; (c) mean zonal velocity $\bar{u}$; (d) maximum zonal velocity *u*_*MAX*_ at the model bottom layer. Unit: ms^-1^. Black dots mark the coral locations. Bathymetric isolines as in Fig 1.

As mention, the only significant difference between the present model configuration and that in [46, 47] was found in the representation of the near bottom velocity extremes. In Fig 3 maps of mean and maximum values of speed ($\sqrt{u^{2}+v^{2}}$) and of the zonal velocity component *u* attained at each near-bottom grid point are presented. Those maps suggested large spatial and temporal variability at the depths of the coral sites. Also, the elevated lateral velocities, up to 0.9 ms^-1^, that have been observed in the vicinity of the Sigsbee Escarpment south of New Orleans in water depths of about 2000 m in association with rapid changes in the bathymetry [65] were captured by ROMS. A number of other local maxima associated with comparable steepness in the bottom slope were present throughout the domain at depths greater than 1500 m. Compared to [46, 47] this solution improved the representation of the maxima in lateral velocities achieved over steep topography at depths greater than 1000 m due to the increased resolution of the bathymetry. For the goal of this work it was important that the model representation of near-bottom velocities, including their magnitude and variability were trustworthy. While the velocity maxima achieved below 1000 m depth are not relevant to the larvae transport, the agreement between model and available observations around and below 1000 m was encouraging and indicates that the simulation captures extreme events in the velocity field.

Only few, sparse in time and space, and usually short time-series of near-bottom velocities have been collected along the continental slope in the GoM, and mostly at depths greater than the coral locations. A mean velocity reconstruction has been attempted using 17 PALACE (Profiling Autonomous Lagrangian Circulation Explorer) floats that mapped the flow at about 900 m of depth between 1998 and 2002 [66]. The mean velocity field derived from these float trajectories agreed with the modeled one and revealed an overall cyclonic pattern with numerous recirculation zones along the continental slope (see Fig 2 in [46]). At depths greater than considered here the modeled lateral velocity field compared favorably to values recorded during the DWH spill [67] and by the Ecosystem Impacts of Oil and Gas Inputs to the Gulf (ECOGIG) consortium (https://ecogig.org) through Acoustic Doppler Current Profilers (ADCPs) and single point current meters deployed on lander systems between 2010 and 2013 in both mean speed and variability (data provided by V. Asper and C. Martens; comparison shown in the Appendix of [46]. Such comparison has been repeated with this model output obtaining similar outcomes).

**Fig 4:**
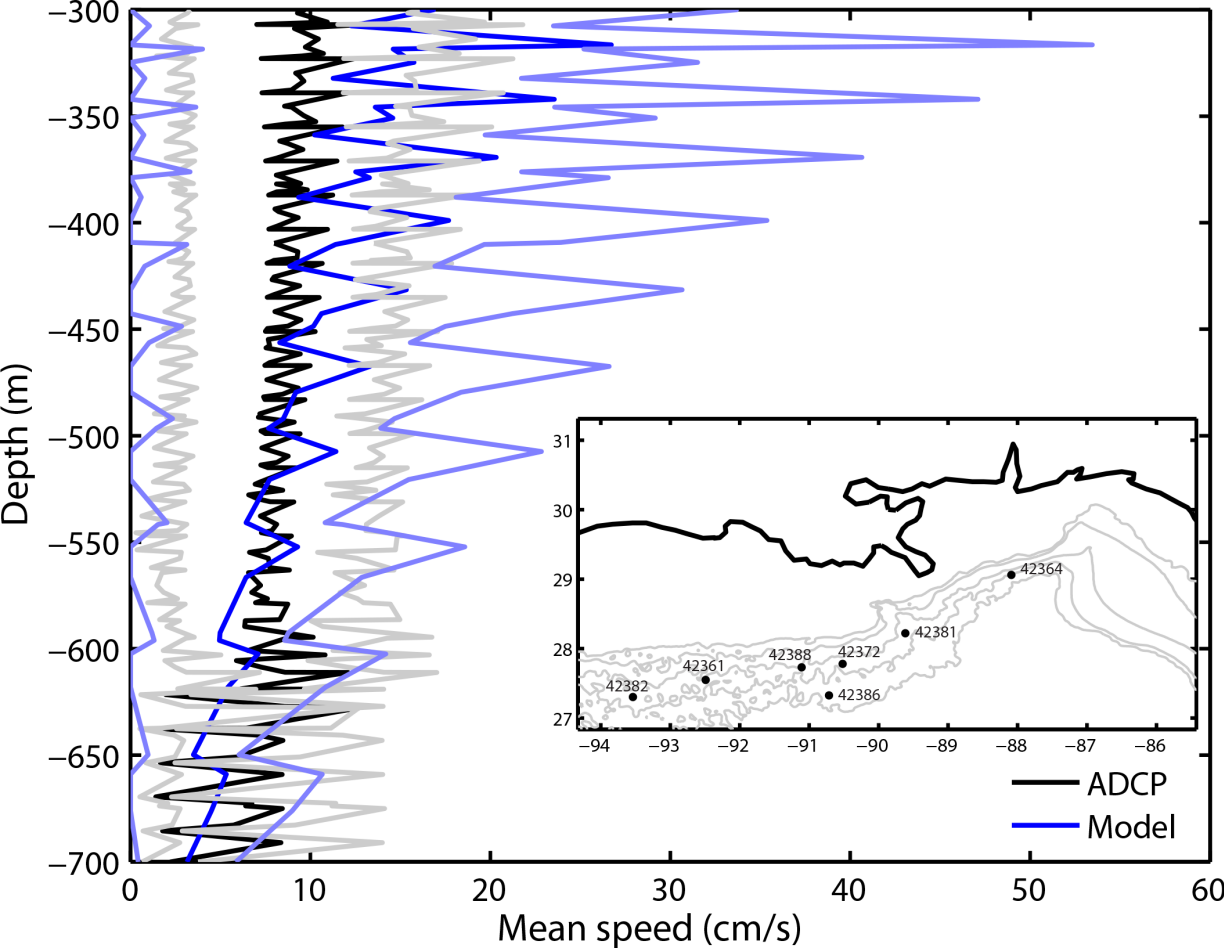
Water column speed. Vertical profile of mean speed (dark blue in the model and black lines in the observations) and standard deviation (light blue in ROMS and gray lines for the ADCP) averaged over the stations and corresponding model grid points shown in the inset.

To our knowledge, reliable data covering the time, depths and geographical areas of the coral sites over the integrated period (2010–2012) are provided only by seven ADCPs deployed at fixed locations near gas and oil off-shore platforms and available through the National Data Buoy Center catalog (http://www.ndbc.noaa.gov/). The time-series at the depths of interest vary in length from eight to twenty-six months and records are not continuous except for the longest (a list of station and their time coverage is available in the Supporting Information, S2 Table). The modeled and observed mean speed and its standard deviations as function of water depth are presented in Fig 4. The modeled curves were calculated using instantaneous velocity values saved every 5 days over three years at the grid points of the ADCP locations at the modeled layer containing the ADCP depths, which are different at each station. The frequency of the ADCP data, on the other hand, is 20 minutes. Considering the much larger number of data points in the ADCP analysis due to the frequency of the recordings and the different spatial coverage (point-wise for the ADCP and a grid by 1 km x 1 km in the model data), we concluded that the modeled mean velocity and standard deviation compared well with the *in-situ* measurements and ROMS correctly reproduced the observed velocity field, while cautioning that velocity data at the depths of the corals are scarce.

### Virtual larvae trajectories

To explore connectivity among coral populations, we released 2,500 neutrally buoyant Lagrangian particles close to the ocean floor four times per year in 2010, 2011 and 2012 (on January 5^th^, March 30^th^, June 30^th^ and September 28^th^) at each of the following locations: VK826 (depth: 600 m), VK906 (depth: 420 m), GB299 (depth: 350 m), and GC140 (depth: 320 m), where colonies of *Leiopathes glaberrima* have been observed.

In all cases the particles were released from an area of 50 km x 50 km centered at the latitudes and longitudes of the VK, GC and GB locations (red squares in Fig 1) and their trajectories were integrated on-line and updated after every 100 seconds, i.e. once per baroclinic time step of the circulation model, in turn chosen to ensure numerical stability and based on the fastest baroclinic wave speed [44]. Sensitivity tests have been performed varying the number of particles over the same deployment regions or considering only 625 tracers in an area 25 km x 25 km centered at the coral locations. The outcome of those tests is presented in the Supporting Information (S3 and S4 Tables).

The Lagrangian tracers adopted in this work are neutrally buoyant and infinitesimally small. Black coral larvae, however, may be slightly negatively buoyant [16, 68] and have a finite size, even if very small compared to the 1 km^2^ grid cell of the model. Particles with buoyancy that differs from the surrounding fluid and have a finite size are subject to a more complex suite of forces than considered here (for the full equation describing their motion, see [69]). A previous work [70] verified that our Lagrangian set-up is relevant to finite size, buoyant larvae by modifying the tracer equation in ROMS to consider small spherical particles up to 10% lighter or heavier than the surrounding fluid. No significant differences between neutrally buoyant and heavy/light particles were found whenever such particles were in presence of a turbulent advective field as in the case in this work.

*L*. *glaberrima* larvae may not diffuse effectively within the water column if they are indeed negatively buoyant. However, larval buoyancy is uncertain and we accounted for the possibility that larvae may diffuse in three releases (in Winter 2011 and 2012, and Fall 2011) where particles were subject to both three dimensional advection and vertical diffusion, using the ‘‘vertical walk” scheme described in [71]. Again sensitivity tests varying the number of particles are included as Supporting Information (S3 Table). Horizontal diffusion was accounted by the parameterization of harmonic mixing of momentum adopted in ROMS (the horizontal velocity field responsible for the horizontal dispersal of the Lagrangian particles is the result of an advective term and a harmonic viscous term).

To account for and explore the dependency of connectivity on competency duration and mortality, we followed the approach outlined in [35] and parameterized the loss of competency with an exponential decay function such that:

$$P^{t+1}=P^{t}e^{-\lambda t}$$ (1)

P^t + 1^ represents the portion of larvae that are competent (i.e. capable of completing settlement and metamorphosis) at time t + 1 and λ is the decay constant (or mortality rate) defined as λ = ln(2)/(PLD/2), where PLD is the pelagic larval duration or maximum competency period.

The paucity of data on *Leiopathes glaberrima* do not allow us to determinate with certainty the length of their competency period. It has been found that for *Antipathes fiodersis* [16], another black coral species, the maximum settlement competency period is around–or at least not significantly longer than– 10 days. However, it has been recently suggested that coral species with lecithotrophic (non-feeding) larvae may be capable of extending their longevity and dispersal potential by several weeks entering a low-metabolism state soon after becoming competent [17], or that their longevity may last as much as three and five weeks, and up to eight weeks under laboratory conditions [18]. We choose to advect the tracers for 40 days, extending by one month the competency period indicated in [16] to include the possibility of a state of low metabolic activity [17], or of greater larval longevity [18]. Here we explore connectivity potentials for PLD/2 = 10, 15, 20, 25 and 30 days. Particle tracking was terminated after 40 days.

The modeled virtual larvae trajectories may be obtained through GRIIDC, doi:10.7266/N7V9860S.

## Results

### Transport analysis

**Fig 5:**
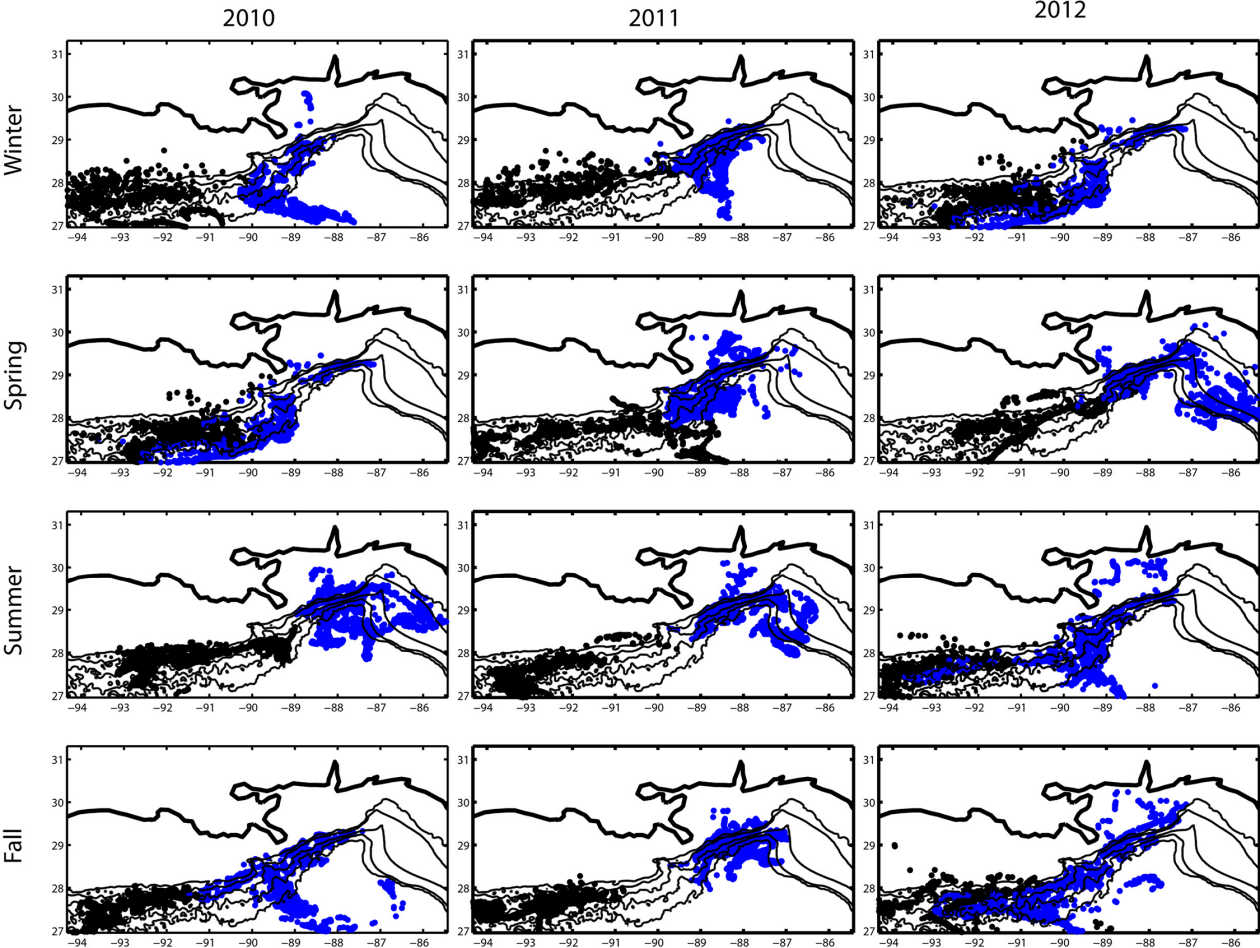
Particles position. Particles position projected onto the horizontal plane 40 days after their release for each deep deployment overlaid bathymetry contours. Particles deployed at VK906 and VK826 are in blue, and particles deployed at GB299 and GC140 are in black.

The direction of motion of the near bottom particles released near the bottom across the four sites was predominantly counterclockwise in winter and fall (VK to GB/GC) and variable in summer and spring. Particles released at the GB/GC sites (black dots in Fig 5) moved mostly along the isobaths in the south-west or north-east direction up to the Mississippi Canyon area, but did not penetrate further east. Particles released at Viosca Knoll (blue particles) moved towards the De Soto Canyon and then off shore predominantly in spring and summer, and mostly westward in the other seasons, reaching the GC area.

**Fig 6:**
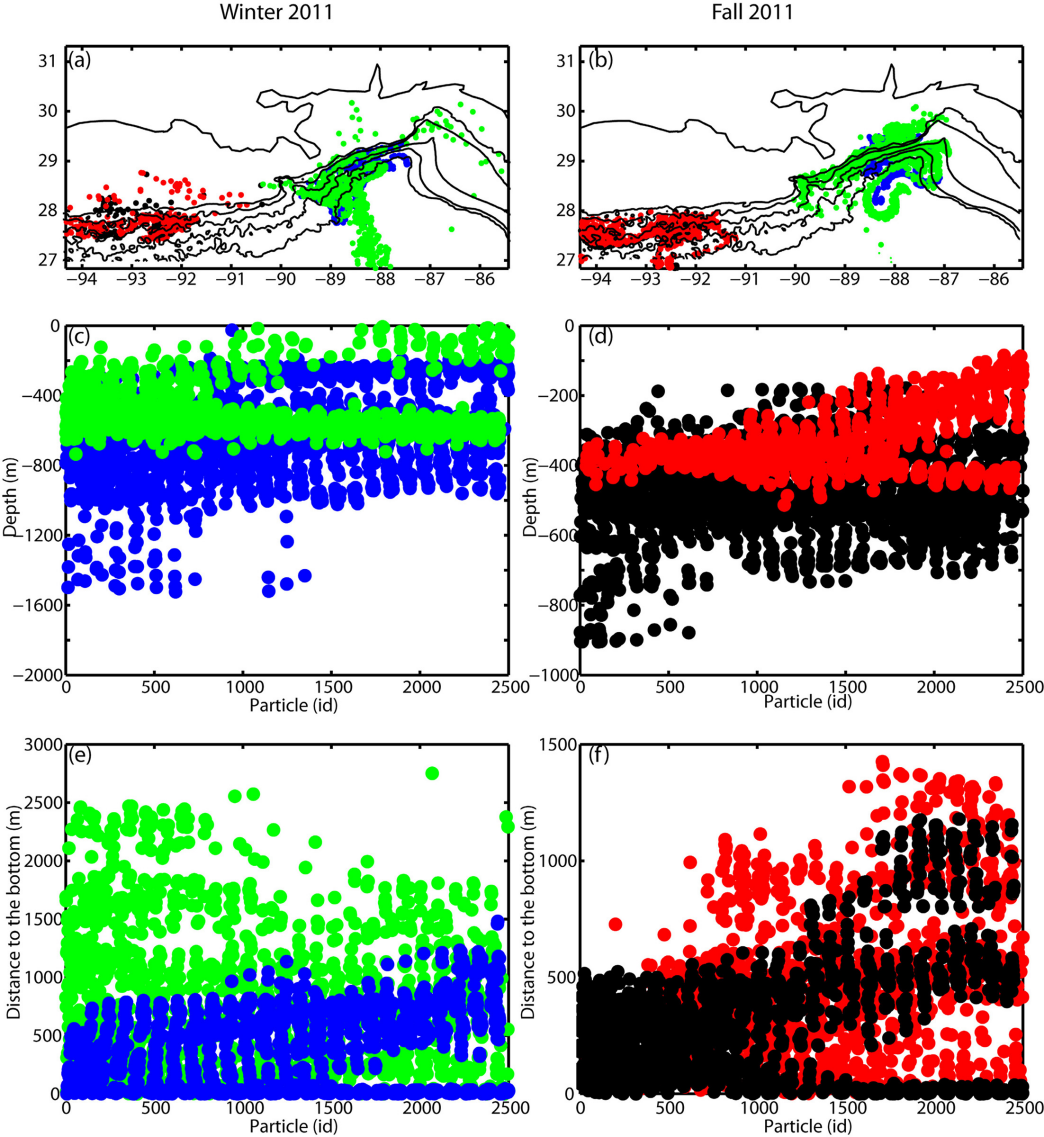
Particle positions, horizontal and vertical, vertical diffusion included. (a)-(b) Particles position 40 days after their release in Winter and Fall 2011 projected onto the horizontal plane. (c)-(d) Depth of particles 40 days after their release plotted against their id tag (from 1 to 2500) and (e)-(f) their distance from the bottom. Particles deployed at VK906 and VK826 are in blue (without vertical diffusion) and green (with vertical diffusion), and particles deployed at GB299 and GC140 are in black (without vertical diffusion) and red (with vertical diffusion).

Particles were free to move in the vertical, but in the absence of a vertical diffusion term the large majority remained confined close to the bottom, following the isobaths and the bathymetric contours as likely to happen due to the negative buoyancy of the larvae (Fig 6). Only particles trapped at the edge of mesoscale eddies displayed large diapycnal excursions [72], giving us confidence in the estimation of connectivity from the location of the tracers in the (x, y) plane. Larvae of *L*. *glaberrima* need to settle on a hard substrate and large vertical excursions may limit their ability to find suitable substrate. The addition of vertical diffusion through a random walk term increased only very moderately the connectivity potential computed using the horizontal coordinates of the final position for any of the cases considered (Table E in S1 Appendix), but displaced a greater number of particles away from the bottom, independently of the release site (Fig 6). Vertical diffusion was added to the 2011 winter and fall, and 2012 winter deployments to test its role in two cases when connectivity is high or when is average compared to other deployments. The virtual larvae covered on average larger distances due to the vertical movement but they were not transported closer to other colonies, while being further away from the bottom substrate. If we had accounted for the z location in the connectivity estimation, the large majority of the particles subject to the vertical diffusion would not satisfy the requirement of being less than 100 m from the bottom.

**Fig 7:**
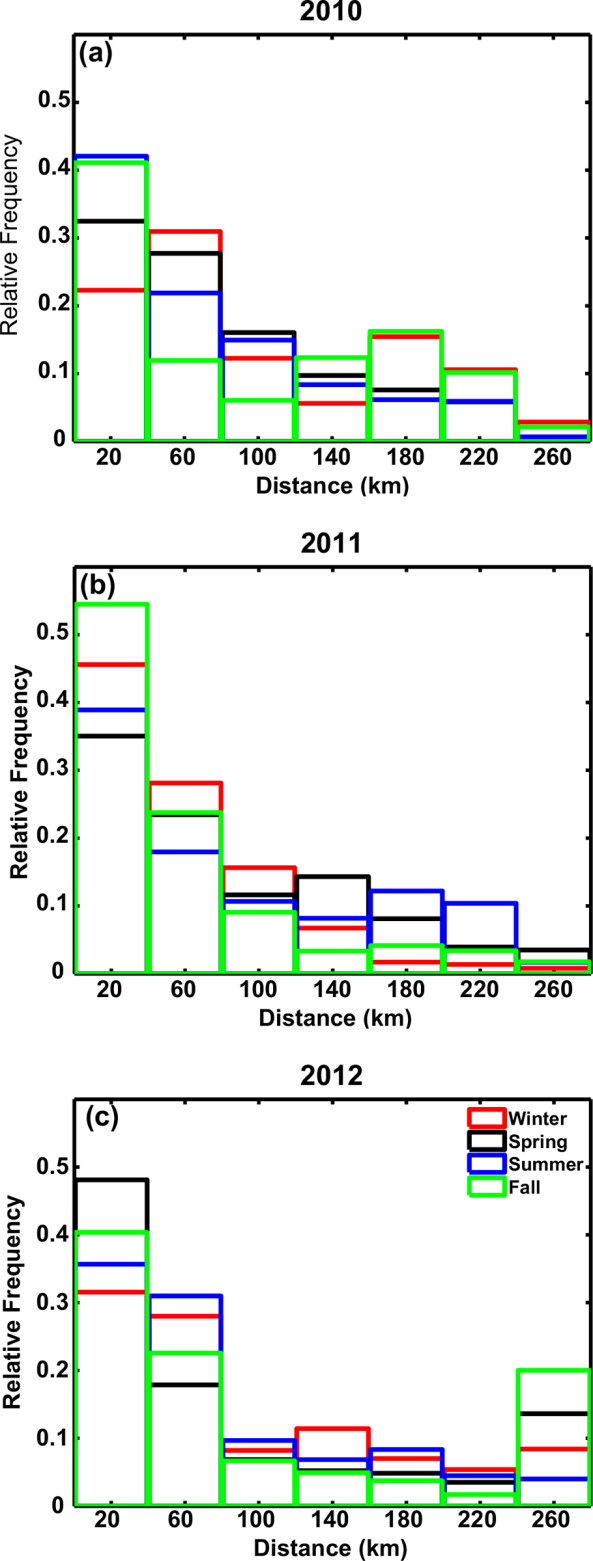
Horizontal distance traveled by particles. Histograms of the horizontal distance traveled by the particles plotted in all seasons. (a) 2010, (b) 2011, and (c) 2012.

The horizontal distance traveled by the particles released at the sea bottom after 40 days did not display any obvious seasonality except for being slightly smaller in summer in all years, but was characterized by interannual variability (Fig 7). In spring, winter and fall 2012, for example, more than twice as many particles as in any other year travelled over 200 km from the release point in response to consistently strong bottom velocities.

### Genetic Analysis

Pairwise comparisons of *L*. *glaberrima* population differentiation corroborated the results of a Bayesian cluster analysis reported in [37]. There was significant overall population differentiation among sites within the northern Gulf of Mexico (average F_st_ = 0.136, p < 0.01). While GC140 and GB299 were not significantly differentiated, each was different from the two Viosca Knoll sites. F_st_ values were 4–6 fold higher between GB299/GC140 and the Viosca Knoll cases than between VK906 and VK826 (Table 1).

**Table 1:** Comparisons of *L*. *glaberrima* population differentiation.

A				
Source of variation	Sum of Squares	Variance components	% variation	
Among populations	101.04	0.48	13.57	
Within populations	822.23	3.07	86.43	
Total	923.27	3.55		
Average Fst				0.136**
B				
	GB299	GC140	VK826	VK906
GB299	0.000			
GC140	0.012	0.000		
VK826	0.169**	0.136**	0.000	
VK906	0.261**	0.212**	0.040**	0.000

*Leiopathes glaberrima* overall (A) and pairwise population differentiation (B) as estimated by an Analysis of Molecular Variance on F_st-_values in the northern Gulf of Mexico. P-values were estimated from 100 permutations and corrected for multiple comparisons (** p < 0.01).

Estimates of migration were calculated with the Migrate-n software using constant mutation rates estimated from the allele frequencies. GC140 and GB299 received the highest number of immigrants per generation (Table 2) when considering both lineages of *L*. *glaberrima* together. Immigration to VK906 was limited, with the exception of immigration from VK826. The direction and magnitude of migration rates generally agrees with Bayesian clustering analyses (Fig 1 in [37]).

**Table 2:** Immigration rates for two sympatric lineages of *L*. *glaberrima* among sites considered together and for Lineage 1 only.

Direction of migration	Migration rates for both lineages	M/4	Immigrants per generation (Theta*M/4)	Migration rates for Lineage 1 only	M/4 for Lineage 1 only	Immigrants per generation (Theta*M/4) for Lineage 1 only
GC140 to GB299	8.23	2.06	3.52	51.44	12.86	1.16
VK826 to GB299	19.93	4.98	8.52	37.69	9.42	0.85
VK906 to GB299	8.23	2.06	3.52	na	na	na
GB299 to GC140	11.85	2.96	1.00	18.13	4.53	0.41
VK826 to GC140	17.72	4.43	1.49	14.45	3.61	0.33
VK906 to GC140	10.31	2.58	0.87	na	na	na
GB299 to VK826	5.08	1.27	0.18	24.02	6.01	0.54
GC140 to VK826	4.83	1.21	0.17	26.05	6.51	0.59
VK906 to VK826	5.80	1.45	0.21	na	na	na
GB299 to VK906	7.77	1.94	0.82	na	na	na
GC140 to VK906	5.95	1.49	0.63	na	na	na
VK826 to VK906	20.40	5.10	2.14	na	na	na

Immigration rates calculated with Migrate-n, using 4 heated chains (starting “temperatures”: 1.0, 1.2, 3.0, 10^6^), and one long chain with 5000 steps and burn-in of 10000. The direction of migration represents the possible origin of immigrants in the current site. Migration rates represent the fraction of individuals in the population that is composed of immigrants. The number of migrants per generation are calculated at Θ *M/4 in which Θ is the mutation-scaled effective population size, and M/4 is the migration rate divided by the inheritance scalar for diploids (4). Separated lineages did not have enough sample size to run the migration analysis for all populations.

Considering lineages of *L*. *glaberrima* separately, GB299 and VK826 received the highest number of immigrants of Lineage 1 per generation (Table 2). VK826 also received the highest number of immigrants per generation of Lineage 2 (Table 3).

**Table 3:** Immigration rates for two sympatric lineages of *L*. *glaberrima* among sites for Lineage 2 only.

Direction of migration	Migration rates for Lineage 2	M/4	Immigrants per generation (Theta*M/4)
VK906 to VK826	15.89	3.97	34.72
VK826 to VK906	16.38	4.10	21.91

Immigration rates calculated with Migrate-n as in Table 2 for Lineage 2. Separated lineages did not have enough sample size to run the migration analysis for all populations.

### Modeled potential connectivity

**Fig 8:**
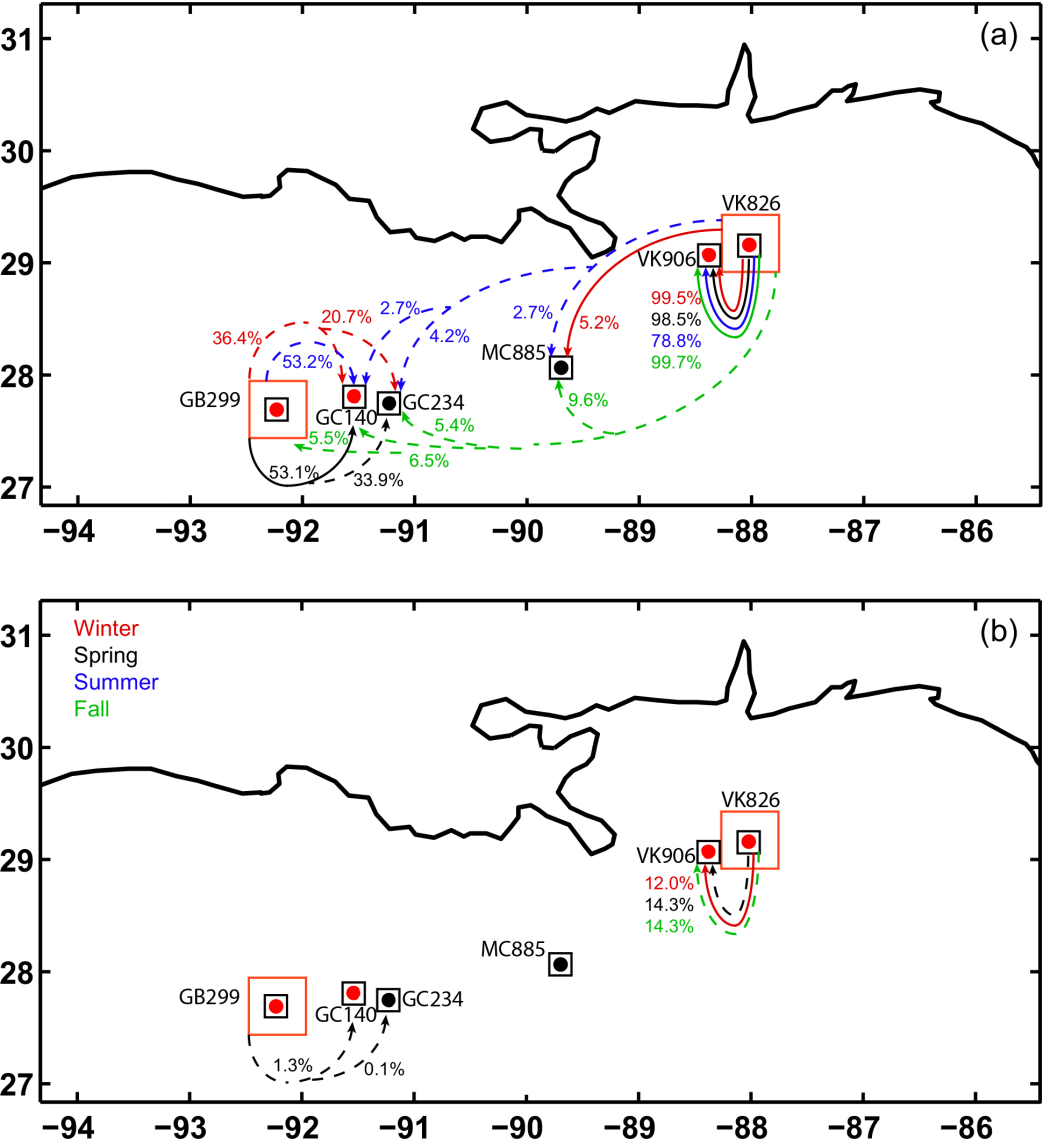
Connectivity diagrams VK826 and GB299. Connectivity diagram for larvae released at VK826 and GB299. (a) No mortality term included. (b) Maximum competency period PLD = 20 days. Solid lines: Locations connected in all years (2010, 2011 and 2012). Dash lines: Locations connected in one or two years. Color indicates season. The percentage marked next to each arrow corresponds to the % of particles that reach the connected site.

**Fig 9:**
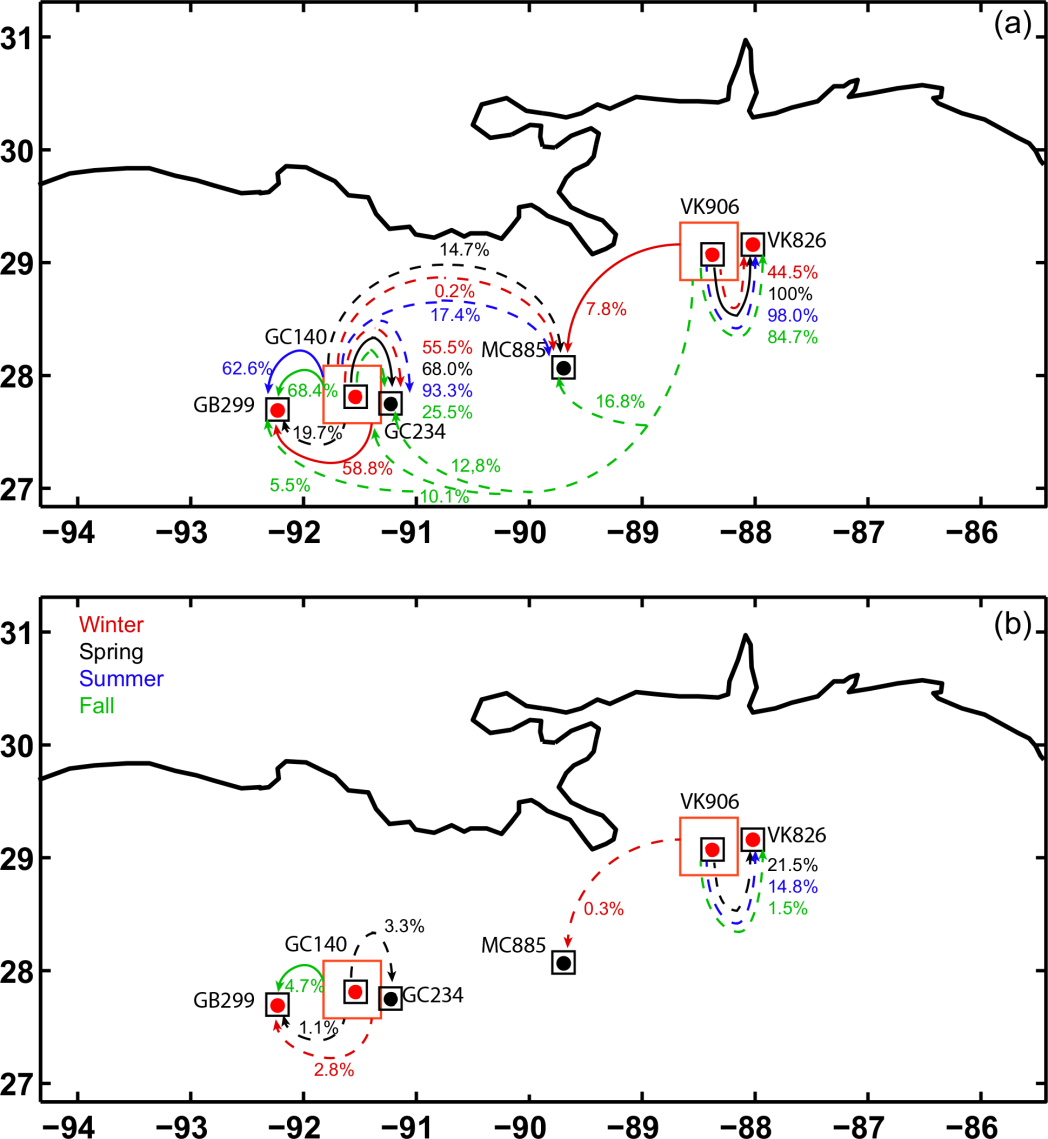
Connectivity diagrams VK906 and GB140. As in Fig 8 but for larvae released at VK906 and GC140.

We explored possible preferential routes for larvae exchanges among sites between the sampled locations as well as two possible stepping stone sites, GC234 (27.75°N, 91.22°W, 520 m) and MC885 (28.06°N, 89.69°W, 650 m) to provide a more comprehensive analysis of potential connectivity. The chosen stepping stone sites are on the upper continental slope near the Mississippi Delta, have a sea floor suitable for coral development, and are populated by *Lophelia pertusa* and other communities dominated by cnidarians [73]. Our deployment areas (50 km x 50 km) largely overestimated the observed area over which the coral colonies occurred. Given the partial superimposition of close-by deployment areas, we limited our targets to squares of size 20 km x 20 km centered at each site (black squares in Figs 8 and 9).

Below we summarize our results in the absence of mortality and for a pelagic larval duration of 20 days. The complete connectivity matrices and all five PLD scenarios, including integrations with vertical diffusion expressed by a random walk, can be found in the S1 Appendix. We focused on the role of seasonality, interannual variability, if any, and of larval life span. Further sensitivity tests (see Supporting Information) show that particle distributions (S1 Fig) or connectivity matrices (S3 and S4 Tables) are robust to changes in the horizontal diffusion coefficient used in ROMS, in the size of the deployment sites, and in the number of particles if such number is sufficiently large.

In pairwise comparisons, the sites at Viosca Knoll (VK906 and VK826) and Green Canyon (GC140 and GC299) were connected with each other, confirming results from the genetic analysis. Even when considering only particles released in the black boxes around each of the sites (i.e. the 400 inner particles), a substantial percentage of larvae reach the other location in all deployments. VK826 and VK906 are about 36 km apart and appeared to be connected during all seasons in the three years considered.

Generally, connectivity was greater from VK826 (shallower) to VK906 (deeper) than vice versa, with the exception of spring 2010, following the preferential mean current direction, again in agreement with the genetic results. If the mortality term was not included, connectivity from VK826 to VK906 was generally highest in fall and winter (as high as 99.7% in fall 2011), and lowest in summer (78.8% or less, depending on the year), but subjected to interannual changes larger than the seasonal fluctuations. Particles released at VK826 can reach VK906 within the first five days, but the connectivity peaked around day 15 in winter and spring, and around day 25 in summer and fall. From VK906 to VK826 the connectivity was, on average, stronger in summer and weaker in winter in the three years considered.

Larvae from Viosca Knoll reached MC885, but connectivity greater than 1% was found only for larvae surviving 20 days or longer. Few particles released at VK826 (between ~ 3% to 5% of the 2,500 deployed) were transported along the continental slope by a topographically intensified current to Green Canyon in summer and fall of 2012, and reached as far as GC140, GC234, and GB299. No connectivity with the sites to the west of the Mississippi mouth was established for particles originating at VK906 or at VK826 whenever a maximum competency period of 30 days or less was included.

Larvae released at GB299 were found only as far as the closest modeled locations GC140 and GC234. When the mortality term was introduced, the connectivity was extremely small and limited to spring 2011.

The connectivity of particles deployed at GC140 extended to GC234 and GB299 with percentages approaching 70% in the absence of mortality and below 10% when competency was introduced as most particles reached the new site after 30 days. The connectivity between GC140 and MC885 was large only in summer 2010 and spring 2012 (about 17 and 15%, respectively). Figs 8 and 9 summarize the analysis for particles without mortality and for a maximum competency period of 20 days.

In summary, strong connectivity was found between VK906/VK826, and among GB299/GC140/GC234. Few particles from VK locations traveled as far as the GC area, following the preferential mean current direction, and none vice versa in the 12 deployments performed. Episodic intensifications of the mean westward current associated with the passage of surface intensified cyclonic mesoscale eddies impinging on the continental slope were responsible for the enhanced transport from VK to GC in 2012. Whenever a maximum competency period of a month or less was introduced, the percentage of larvae capable of reaching other colonies decreased noticeably, and the connectivity was generally limited to the nearest site. The connectivity around the GB and GC area was stronger in winter and spring, compared to summer and fall, while around Viosca Knoll it was greater in fall and winter compared to summer. Large interannual changes in connectivity were found at all sites.

## Discussion

Deep-sea ecosystems such as those in the northern Gulf of Mexico are increasingly subjected to anthropogenic disturbance because of their proximity to oil and mineral deposits [16]. They also occur in a seascape of hard substrates separated by large distances and are subjected to minimal physical variability in comparison to wave-battered shallow-water ecosystems [74–76]. Information about their scale of dispersal via their planktonic larvae is therefore essential to understand how populations expand to new habitat and recover from natural or anthropogenic disturbances [35, 77–79].

Here, we used a hydrodynamical circulation model of larval dispersal to show that a habitat-forming black coral, *Leiopathes glaberrima*, rarely disperses between two regions in the northern Gulf of Mexico, separated by less than 500 km, and that of the four sites investigated VK906 experienced the lowest levels of immigration and emigration except than with its neighboring site, VK826. This behavior was associated, in the model, with weak near-bottom shelf currents–a characteristic common to many deep sites–and with the presence of numerous instabilities generated by the current passing over the complicated bathymetry of the Gulf of Mexico. Those mesoscale and submesoscale instabilities are responsible for frequent changes in the transport direction, detected in our 1 km horizontal resolution runs.

We found that vertical migration associated with physical advection was limited to 50 m from the bottom on average, and only a dozen particles deployed at depth reached the surface in our simulations in the absence of an explicit vertical diffusion term. Coral larvae are weak swimmers [68, 80] however we cannot exclude that they might be able to determine their direction of travel by migrating vertically as other invertebrate and vertebrate larvae do [81, 82]. We do not know how fast black coral larvae can swim or if they can change their buoyancy in their lifespan, and we did not account for these possibilities, but the vertical distance they need to cover to reach the Ekman layer, where lateral currents are strong, is large (> 200 meters) in light of their potentially negative buoyancy [68]. Furthermore, the addition of a vertical diffusion term in form of a random walk did not improve the modeled connectivity potential.

The outcome of the hydrodynamical model was in general agreement with estimates of low realized connectivity from multi-locus genetic data, as indicated in Table 1. The observation that colonies of *Leiopathes glabberima* to the east and west of the Mississippi Canyon are only rarely connected via larval dispersal suggests that there is a physical barrier to fast dispersal between coral colonies at Viosca Knoll and Green Canyon and depends, in the model, on the details of the bathymetry and on assumptions on the life span of the larvae. Our analysis showed that even assuming competency for 40 days, very few virtual larvae dispersed successfully between sites in both parent and nested runs, and suggested that a longevity of a month or longer is required for the larvae of *Leiopathes glaberrima* to achieve the exchange rates measured in the genetic analysis.

The broad agreement between genetic and biophysical models suggests that dispersal limitation played some role in structuring populations of *L*. *glaberrima* in the northern Gulf of Mexico. Note however that the genetic data integrates over approximately 100s to 10,000s of generations of successful gene flow events whereas the biophysical model was forced by just three years of data. If one generation per year is assumed then the genetic data may span the period between the last glacial maximum and present. During this time window the mean circulation in the northern Gulf of Mexico did not change significantly despite morphodynamic changes in the bathymetry due to erosion and deposition, changes in sea level, changes in the extension of the shallower (less than 50 m) portion of the shelf due to deformation from the ice-sheet relevant between 10,000 and 5,000 years ago, and a progradation of the area around Mississippi Delta of about 100 km between 5,000 years ago and present [83]. The exact dispersal percentages from the biophysical and the genetic model did not coincide and they were not expected to given the difference in the time horizon, and hence we restricted the comparison between models to a discussion of general dispersal trends.

Larvae dispersal is influenced not only by pre-settlement processes (competency, duration and mortality), and by physical processes (currents, eddies, fronts), but also by post-settlement mortality [81,84,85]. Hence, even if larvae might disperse between sites [86], they might not survive, or survive in one direction but not the other [87]. With respect to the physical forcing, not all processes may be included even at the resolution of 1 km. Bathymetry details are especially important and have been shown to be linked to the occurrence of difference color morphs of *L*. *glaberrima* [35], but are not fully resolved here. Further increases in horizontal resolution would require using hydrostatic ocean circulation models and would increase significantly the computational costs.

Black corals are extremely long-lived [88, 89] and hence once they colonize a site they tend to settle for long times. According to our model the stable physical environment of the northern Gulf favors local recruitment with only occasional long-distance dispersal, creating a series of more-or-less separate local populations linked by relatively low rates of gene flow [90]. The subdivision of a widespread taxon into discrete populations that function as separate ecological units has important implications. In terms of management, it implies that any perturbation to a local system will have primarily local consequences, but that recovery may be delayed because of low immigration of conspecifics from other areas. In terms of conservation priorities, the spatial heterogeneity may cause critical genetic variations, such that the loss of any single population could reduce genetic diversity in the taxon overall [90]. More specifically to the *Leiopathes glaberrima* in the Gulf of Mexico, the proximity to natural oil deposits is one of the greatest threats [91]. Because this species is so long-lived and slow growing, and because sexual recruitment rates are extremely low in deep-water corals [30, 92], combined with limited dispersal among sites [37], any recovery most likely will depend on local larvae produced by surviving individuals.

In this work we have shown that using jointly biophysical and genetic models can contribute insights and allow for hypothesis testing about dispersal processes even for remote ecosystems. Most published work on the topic has focused on shallow-water systems [35, 79, 93]. Our results indicate that deep currents can be characterized by substantial spatial and temporal variability that prevents dispersal between relatively close-by sites, and that biophysical models can provide useful information to help interpret genetic analyses.

## Supporting Information

S1 Appendix Connectivity matrices. (DOCX)

S1 Fig Horizontal viscosity coefficient sensitivity test. Top: Modeled zonal (west to east) velocity field on February 3^rd^, 2011 obtained setting the horizontal harmonic mixing coefficient A_h_ equal to a) 5 m s^-2^ and b) 25 m s^-2^. Bottom: particle distributions c) 10 days and d) 40 days past deployment. Black and blue particles: A_h_ = 5 m s^-2^; red and green particles: A_h_ = 25 m s^-2^.
(PDF)

S1 Table Migration rates calculated with BayesAss. Immigration rates calculated with BayesAss, using random seed determined by the program, the Markov Chain Monte Carlo was run for 10 million iterations discarding the first 1 million iterations burn-in = 1000000), with and interval within MCMC samples of 1000. The direction of migration represents the possible origin of immigrants in the current site. Migration rates represent the fraction of individuals in the population that is composed of immigrants. S.D. = Standard Deviation. Separated lineages did not have enough sample size to run the migration analysis for all the populations where they were found.
(PDF)

S2 Table ADCP data availability from off-shore oil platforms. List of platform stations and their time availability (gray boxes) used in the model validation process. Data are from the National Data Buoy Center catalog (http://www.ndbc.noaa.gov/).
(PDF)

S3 Table **Sensitivity tests (part 1).** Tests varying the number of particles, deployment area extension, and the inclusion of vertical diffusion scheme for winter 2011, fall 2011 and winter 2012. **VD**: Vertical Diffusion; **A1**: Deployment area 50 km x 50 km; **A2**: Deployment area 25 km x 25 km.
(PDF)

S4 Table **Sensitivity test (Part 2).** Sensitivity tests varying the number of particles for an identical deployment area extension (50 km x 50 km) and no vertical diffusion for winter 2011, fall 2011 and winter 2012. 100, 1000 and 2500 particles.
(PDF)
