# Supplementary material for: Potential Connectivity of Coldwater Black Coral Communities in the Northern Gulf of Mexico
Source: PLoS One. 2016 May 24;11(5):e0156257. doi: 10.1371/journal.pone.0156257 (PMC4878809; doi:10.1371/journal.pone.0156257)
Supplement: S1 Appendix — (DOCX) [file pone.0156257.s001.docx]

**S1 Appendix. Connectivity matrices**

The connectivity matrices calculated on the based of the physical model results are presented in the following Tables A-D. The matrices are organized by season in Table A-D and include vertical diffusion for the three experiments repeated with the addition of a random walk along *z* in Table E. The percentage of particles that reaches any other site (when greater than zero) and the day of first arrival are indicated.

**Table A.** **Connectivity matrix for winter.**

| **PLD**  **days** | **Winter** | **VK826 to VK906** | | **VK826 to MC885** | | **GB299 to GC140** | | **GB299 to GC234** | | **GC140 to GB299** | | **GC140 to GB234** | | **GC140 to MC885** | | **VK906 to VK826** | | **VK906 to MC885** | |
| --- | --- | --- | --- | --- | --- | --- | --- | --- | --- | --- | --- | --- | --- | --- | --- | --- | --- | --- | --- |
|  |  | **day** | **%** | **day** | **%** | **day** | **%** | **day** | **%** | **day** | **%** | **day** | **%** | **day** | **%** | **day** | **%** | **day** | **%** |
| No PLD | 2010 | 4 | 96.5 | 17 | 5.2 |  |  |  |  | 10 | 54.6 | 28 | 55.5 |  |  |  |  | 23 | 1.2 |
|  | 2011 | 4 | 99.5 | 18 | 0.2 |  |  |  |  | 5 | 58.8 | 9 | 18.7 | 29 | 0.2 |  |  | 17 | 4.7 |
|  | 2012 | 4 | 63.5 | 24 | 0.4 | 11 | 36.4 | 13 | 20.7 | 5 | 9.2 | 17 | 86.7 |  |  | 7 | 44.5 | 7 | 7.8 |
| 30 | 2010 | 4 | 9.0 |  |  |  |  |  |  | 7 | 4 |  |  |  |  |  |  |  |  |
|  | 2011 | 4 | 15 |  |  |  |  |  |  | 5 | 6.9 | 9 | 1.0 |  |  |  |  |  |  |
|  | 2012 | 4 | 13.5 |  |  | 11 | 0.1 |  |  | 5 | 1.0 |  |  |  |  |  |  | 5 | 0.7 |
| 25 | 2010 | 4 | 7.5 |  |  |  |  |  |  |  |  |  |  |  |  |  |  |  |  |
|  | 2011 | 4 | 14.5 |  |  |  |  |  |  | 5 | 4.3 |  |  |  |  |  |  |  |  |
|  | 2012 | 4 | 10.0 |  |  |  |  |  |  | 5 | 0.8 |  |  |  |  |  |  | 7 | 0.5 |
| 20 | 2010 | 4 | 7.0 |  |  |  |  |  |  |  |  |  |  |  |  |  |  |  |  |
|  | 2011 | 4 | 12.0 |  |  |  |  |  |  | 5 | 2.8 |  |  |  |  |  |  |  |  |
|  | 2012 | 4 | 7.8 |  |  |  |  |  |  | 5 | 0.4 |  |  |  |  |  |  | 7 | 0.3 |
| 15 | 2010 | 5 | 4.7 |  |  |  |  |  |  |  |  |  |  |  |  |  |  |  |  |
|  | 2011 | 4 | 5.5 |  |  |  |  |  |  | 5 | 1.4 |  |  |  |  |  |  |  |  |
|  | 2012 | 4 | 5.0 |  |  |  |  |  |  | 5 | 0.5 |  |  |  |  |  |  | 7 | 0.2 |
| 10 | 2010 | 5 | 1.3 |  |  |  |  |  |  |  |  |  |  |  |  |  |  |  |  |
|  | 2011 | 4 | 1.5 |  |  |  |  |  |  | 5 | 0.5 |  |  |  |  |  |  |  |  |
|  | 2012 | 4 | 2.5 |  |  |  |  |  |  | 5 | 0.1 |  |  |  |  |  |  |  |  |

Connectivity matrix for winter deployments (releases on January 5^th^ in each year). Overall percentage of particles reaching any other site (when greater than zero) and the day of first arrival are indicated.

**Table B.** **Connectivity matrix for spring.**

| **PLD**  **days** | **Spring** | **VK826 to VK906** | | **GB299 to GC140** | | **GB299 to GC234** | | **GB299 to MC885** | | **GC140 to GB299** | | **GC140 to GB234** | | **GC140 to MC885** | | **VK906 to VK826** | |
| --- | --- | --- | --- | --- | --- | --- | --- | --- | --- | --- | --- | --- | --- | --- | --- | --- | --- |
|  |  | **day** | **%** | **day** | **%** | **day** | **%** | **day** | **%** | **day** | **%** | **day** | **%** | **day** | **%** | **day** | **%** |
| No PLD | 2010 | 13 | 34.0 | 27 | 2.2 |  |  |  |  | 4 | 19.7 | 24 | 10 |  |  | 4 | 100 |
|  | 2011 | 4 | 98.0 | 5 | 15.2 | 6 | 1.4 | 14 | 12.3 | 19 | 13.3 | 4 | 45.7 | 16 | 11.9 | 13 | 5.5 |
|  | 2012 | 11 | 98.5 | 18 | 53.1 | 22 | 34.0 | 28 | 10.3 |  |  | 18 | 68.0 | 29 | 14.7 | 6 | 36.5 |
| 30 | 2010 |  |  |  |  |  |  |  |  | 4 | 1.2 |  |  |  |  | 4 | 31.5 |
|  | 2011 | 4 | 21.3 | 5 | 3.2 | 5 | 0.2 |  |  |  |  | 4 | 5.3 |  |  |  |  |
|  | 2012 |  |  |  |  |  |  |  |  |  |  |  |  |  |  |  |  |
| 25 | 2010 |  |  |  |  |  |  |  |  | 4 | 1.2 |  |  |  |  | 4 | 27.2 |
|  | 2011 | 4 | 16.2 | 5 | 2.3 | 6 | 0.2 |  |  |  |  | 4 | 4.7 |  |  |  |  |
|  | 2012 |  |  |  |  |  |  |  |  |  |  |  |  |  |  |  |  |
| 20 | 2010 |  |  |  |  |  |  |  |  | 4 | 1.1 |  |  |  |  | 4 | 21.5 |
|  | 2011 | 4 | 14.2 | 5 | 1.4 | 6 | 0.1 |  |  |  |  | 4 | 3.2 |  |  |  |  |
|  | 2012 |  |  |  |  |  |  |  |  |  |  |  |  |  |  |  |  |
| 15 | 2010 |  |  |  |  |  |  |  |  | 4 | 0.6 |  |  |  |  | 4 | 14.2 |
|  | 2011 | 4 | 6.5 | 5 | 0.6 |  |  |  |  |  |  | 4 | 2.2 |  |  |  |  |
|  | 2012 |  |  |  |  |  |  |  |  |  |  |  |  |  |  |  |  |
| 10 | 2010 |  |  |  |  |  |  |  |  | 4 | 0.2 |  |  |  |  | 4 | 6.7 |
|  | 2011 | 4 | 3.2 | 5 | 0.2 |  |  |  |  |  |  | 4 | 1.5 |  |  |  |  |
|  | 2012 |  |  |  |  |  |  |  |  |  |  |  |  |  |  |  |  |

As in Table A but for spring deployments (releases on March 30^th^ in each year).

**Table C**. **Connectivity matrix for summer.**

| **PLD**  **days** | **Summer** | **VK826 to VK906** | | **VK826 to MC885** | | **VK826 to GC234** | | **VK826 to GC140** | | **GC140 to GB299** | | **GC140 to MC885** | | **VK906 to VK826** | |
| --- | --- | --- | --- | --- | --- | --- | --- | --- | --- | --- | --- | --- | --- | --- | --- |
|  |  | **day** | **%** | **day** | **%** | **day** | **%** | **day** | **%** | **day** | **%** | **day** | **%** | **day** | **%** |
| No PLD | 2010 | 18 | 31.5 |  |  |  |  |  |  | 19 | 19.6 | 4 | 93.2 | 18 | 17.4 |
|  | 2011 | 18 | 69.5 |  |  |  |  |  |  | 9 | 62.6 |  |  |  |  |
|  | 2012 | 4 | 78.7 | 16 | 2.7 | 20 | 4.2 | 23 | 2.7 | 25 | 39.6 |  |  |  |  |
| 30 | 2010 |  |  |  |  |  |  |  |  |  |  | 4 | 20.2 |  |  |
|  | 2011 |  |  |  |  |  |  |  |  |  |  |  |  |  |  |
|  | 2012 | 4 | 3.2 |  |  |  |  |  |  |  |  |  |  |  |  |
| 25 | 2010 |  |  |  |  |  |  |  |  |  |  | 4 | 19.0 |  |  |
|  | 2011 |  |  |  |  |  |  |  |  |  |  |  |  |  |  |
|  | 2012 | 4 | 2.0 |  |  |  |  |  |  |  |  |  |  |  |  |
| 20 | 2010 |  |  |  |  |  |  |  |  |  |  | 4 | 12.7 |  |  |
|  | 2011 |  |  |  |  |  |  |  |  |  |  |  |  |  |  |
|  | 2012 | 4 | 0.7 |  |  |  |  |  |  |  |  |  |  |  |  |
| 15 | 2010 |  |  |  |  |  |  |  |  |  |  | 4 | 7.7 |  |  |
|  | 2011 |  |  |  |  |  |  |  |  |  |  |  |  |  |  |
|  | 2012 | 4 | 0.5 |  |  |  |  |  |  |  |  |  |  |  |  |
| 10 | 2010 |  |  |  |  |  |  |  |  |  |  | 4 | 4.5 |  |  |
|  | 2011 |  |  |  |  |  |  |  |  |  |  |  |  |  |  |
|  | 2012 | 4 | 0.3 |  |  |  |  |  |  |  |  |  |  |  |  |

As in Table A but for summer deployments (releases on June 30^th^ in each year).

**Table D.** **Connectivity matrix for fall.**

| **PLD**  **days** | **Fall** | **VK826 to VK906** | | **VK826 to MC885** | | **VK826 to GC234** | | **VK826 to GC140** | | **GC140 to GB299** | | **GC140 to GB234** | | **VK906 to VK826** | | **VK906 to MC885** | |
| --- | --- | --- | --- | --- | --- | --- | --- | --- | --- | --- | --- | --- | --- | --- | --- | --- | --- |
|  |  | **day** | **%** | **day** | **%** | **day** | **%** | **day** | **%** | **day** | **%** | **day** | **%** | **day** | **%** | **day** | **%** |
| No PLD | 2010 | 4 | 95.7 | 20 | 0.4 |  |  |  |  | 8 | 68.4 |  |  | 11 | 84.7 |  |  |
|  | 2011 | 4 | 99.7 |  |  |  |  |  |  | 4 | 49.8 | 8 | 25.5 | 7 | 54.5 |  |  |
|  | 2012 | 4 | 99.3 | 13 | 9.6 | 20 | 5.4 | 24 | 6.5 | 6 | 56.9 | 18 | 6.5 |  |  | 11 | 16.8 |
| 30 | 2010 | 4 | 20.5 |  |  |  |  |  |  | 8 | 1.2 |  |  |  |  |  |  |
|  | 2011 | 6 | 3.7 |  |  |  |  |  |  | 4 | 9.6 | 11 | 0.7 | 8 | 4.0 |  |  |
|  | 2012 | 4 | 20.5 |  |  |  |  |  |  | 5 | 6.2 |  |  |  |  |  |  |
| 25 | 2010 | 4 | 17.0 |  |  |  |  |  |  | 8 | 0.7 |  |  |  | 5 |  |  |
|  | 2011 |  |  |  |  |  |  |  |  | 4 | 7.0 |  |  | 8 | 2.2 |  |  |
|  | 2012 | 4 | 15.2 |  |  |  |  |  |  | 5 | 4.6 |  |  |  |  |  |  |
| 20 | 2010 | 4 | 14.2 |  |  |  |  |  |  |  |  |  |  |  |  |  |  |
|  | 2011 |  |  |  |  |  |  |  |  | 4 | 4.7 |  |  | 8 | 1.5 |  |  |
|  | 2012 | 4 | 10.5 |  |  |  |  |  |  | 4 | 2.1 |  |  |  |  |  |  |
| 15 | 2010 | 4 | 7.7 |  |  |  |  |  |  |  |  |  |  |  |  |  |  |
|  | 2011 |  |  |  |  |  |  |  |  | 4 | 2.88 |  |  | 8 | 0.5 |  |  |
|  | 2012 | 4 | 7.2 |  |  |  |  |  |  |  |  |  |  |  |  |  |  |
| 10 | 2010 | 4 | 4.5 |  |  |  |  |  |  |  |  |  |  |  |  |  |  |
|  | 2011 |  |  |  |  |  |  |  |  | 4 | 1.0 |  |  |  |  |  |  |
|  | 2012 | 4 | 3.7 |  |  |  |  |  |  | 5 | 0.2 |  |  |  |  |  |  |
| No  PLD | 2012 | **VK906 to GC234** | | 20 | 12.8 | **VK906 to GC140** | | 21 | 10.1 | **VK906 to GC299** | | 24 | 5.5 |  |  |  |  |

As in Table A but for fall deployments (releases on September 28^th^ in each year).

**Table E.** **Connectivity matrix in presence of vertical diffusion.**

| **PLD**  **days** | **Season** | | **VK826 to VK906** | | **VK826 to MC885** | | **VK826 to GC234** | | **VK826 to GC140** | | **VK826 to GB299** | | **GB299to GC140** | | **GB299to GC140** | |
| --- | --- | --- | --- | --- | --- | --- | --- | --- | --- | --- | --- | --- | --- | --- | --- | --- |
|  |  |  | **day** | **%** | **day** | **%** | **day** | **%** | **day** | **%** | **day** | **%** | **day** | **%** | **day** | **%** |
| No PLD | Winter | 2011 | 4 | 68 | 18 | 1.7 | 18 | 0.6 | 21 | 0.7 | 14 | 3.36 |  |  |  |  |
|  |  | 2012 | 4 | 22.8 | 4 | 18.6 |  |  |  |  |  |  | 8 | 55.6 | 9 | 42.8 |
|  | Fall | 2011 | 3 | 100 |  |  |  |  |  |  |  |  |  |  |  |  |
| 30 | Winter | 2011 | 4 | 16.5 |  |  |  |  |  |  |  |  |  |  |  |  |
|  |  | 2012 | 4 | 18.5 | 5 | 0.3 |  |  |  |  |  |  | 8 | 0.1 |  |  |
|  | Fall | 2011 | 3 | 1.8 |  |  |  |  |  |  |  |  |  |  |  |  |
| 25 | Winter | 2011 | 4 | 18.0 |  |  |  |  |  |  |  |  |  |  |  |  |
|  |  | 2012 | 4 | 12.5 | 5 | 0.24 |  |  |  |  |  |  | 8 | 0.1 |  |  |
|  | Fall | 2011 | 9 | 0.3 |  |  |  |  |  |  |  |  |  |  |  |  |
| 20 | Winter | 2011 | 4 | 10.3 |  |  |  |  |  |  |  |  |  |  |  |  |
|  |  | 2012 | 4 | 7.8 | 7 | 0.2 |  |  |  |  |  |  |  |  |  |  |
|  | Fall | 2011 | 3 | 0.3 |  |  |  |  |  |  |  |  |  |  |  |  |
| 15 | Winter | 2011 | 4 | 7.5 |  |  |  |  |  |  |  |  |  |  |  |  |
|  |  | 2012 | 4 | 3.0 |  |  |  |  |  |  |  |  |  |  |  |  |
|  | Fall | 2011 |  |  |  |  |  |  |  |  |  |  |  |  |  |  |
| 10 | Winter | 2011 | 4 | 3.8 |  |  |  |  |  |  |  |  |  |  |  |  |
|  |  | 2012 |  |  |  |  |  |  |  |  |  |  |  |  |  |  |
|  | Fall | 2011 |  |  |  |  |  |  |  |  |  |  |  |  |  |  |

As in Table A but for deployments in winter 2011, 2012 and fall 2011 in presence of vertical diffusion.
