# Supplementary figures and images for: Potential Connectivity of Coldwater Black Coral Communities in the Northern Gulf of Mexico
Source: PLoS One. 2016 May 24;11(5):e0156257. doi: 10.1371/journal.pone.0156257 (PMC4878809; doi:10.1371/journal.pone.0156257)

(a)  $u$  (m/s)  $\text{visc}=5 \text{ m}^2/\text{s}$

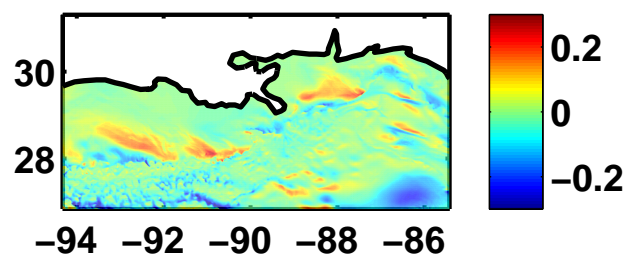

(b)  $u$  (m/s)  $\text{visc}=25 \text{ m}^2/\text{s}$

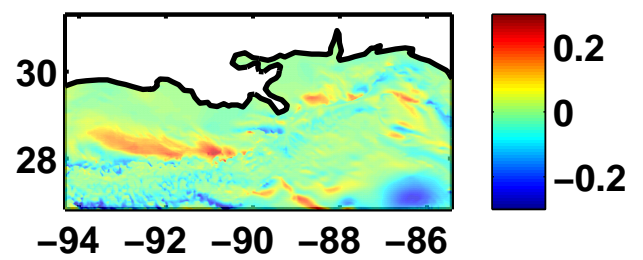

(c) Day 10

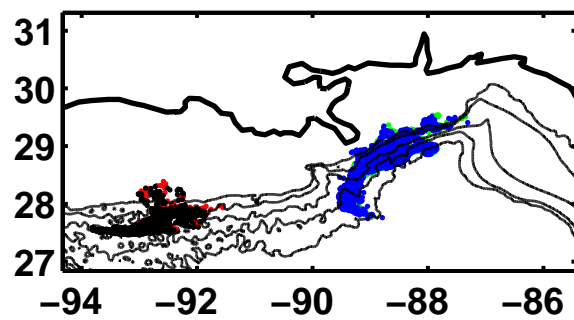

(d) Day 40

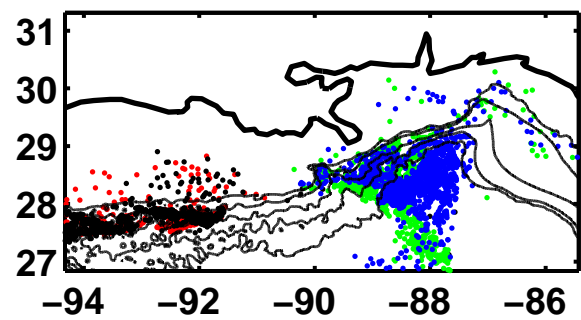

Supplement: S1 Fig — Top: Modeled zonal (west to east) velocity field on February 3rd, 2011 obtained setting the horizontal harmonic mixing coefficient Ah equal to a) 5 m s-2 and b) 25 m s-2. Bottom: particle distributions c) 10 days and d) 40 days past deployment. Black and blue particles: Ah = 5 m s-2; red and green particles: Ah = 25 m s-2. (PDF) [file pone.0156257.s002.pdf]
