# Supplementary material for: Potential Connectivity of Coldwater Black Coral Communities in the Northern Gulf of Mexico
Source: PLoS One. 2016 May 24;11(5):e0156257. doi: 10.1371/journal.pone.0156257 (PMC4878809; doi:10.1371/journal.pone.0156257)
Supplement: S1 Table — Immigration rates calculated with BayesAss, using random seed determined by the program, the Markov Chain Monte Carlo was run for 10 million iterations discarding the first 1 million iterations burn-in = 1000000), with and interval within MCMC samples of 1000. The direction of migration represents the possible origin of immigrants in the current site. Migration rates represent the fraction of individuals in the population that is composed of immigrants. S.D. = Standard Deviation. Separated lineages did not have enough sample size to run the migration analysis for all the populations where they were found. (PDF) [file pone.0156257.s003.pdf]

	Migration rate L1	S.D.	Migration rate L2	S.D.
M GB299 to GB299	0.96	0.02		
M GB299 to GC140	0.02	0.01		
M GB299 to VK826	0.01	0.01		
M GB299 to VK906	0.01	0.01		
M GC140 to GB299	0.29	0.02		
M GC140 to GC140	0.68	0.01		
M GC140 to VK826	0.01	0.01		
M GC140 to VK906	0.01	0.01		
M VK826 to GB299	0.26	0.04		
M VK826 to GC140	0.03	0.02		
M VK826 to VK826	0.69	0.02	0.89	0.06
M VK826 to VK906	0.02	0.02	0.11	0.06
M VK906 to GB299	0.13	0.07		
M VK906 to GC140	0.07	0.06		
M VK906 to VK826	0.07	0.06	0.09	0.09
M VK906 to VK906	0.73	0.05	0.91	0.09
