# Supplementary material for: Potential Connectivity of Coldwater Black Coral Communities in the Northern Gulf of Mexico
Source: PLoS One. 2016 May 24;11(5):e0156257. doi: 10.1371/journal.pone.0156257 (PMC4878809; doi:10.1371/journal.pone.0156257)
Supplement: S2 Table — List of platform stations and their time availability (gray boxes) used in the model validation process. Data are from the National Data Buoy Center catalog (http://www.ndbc.noaa.gov/). (PDF) [file pone.0156257.s004.pdf]

		Platform						
		42372	42381	42382	42386	42388	42361	42364
2010	J							
	F							
	M							
	A							
	M							
	J							
	J							
	A							
	S							
	O							
	N							
	D							
2011	J							
	F							
	M							
	A							
	M							
	J							
	J							
	A							
	S							
	O							
	N							
	D							
2012	J							
	F							
	M							
	A							
	M							
	J							
	J							
	A							
	S							
	O							
	N							
	D							
