# Supplementary material for: Potential Connectivity of Coldwater Black Coral Communities in the Northern Gulf of Mexico
Source: PLoS One. 2016 May 24;11(5):e0156257. doi: 10.1371/journal.pone.0156257 (PMC4878809; doi:10.1371/journal.pone.0156257)
Supplement: S4 Table — Sensitivity test (Part 2). Sensitivity tests varying the number of particles for an identical deployment area extension (50 km x 50 km) and no vertical diffusion for winter 2011, fall 2011 and winter 2012. 100, 1000 and 2500 particles. (PDF) [file pone.0156257.s006.pdf]

Winter 2011						
	VK826 to VK906			VK826 to MC885		
# particles	2500	1000	100	2500	1000	100
No PLD	100	95.4	99	0.2	0.2	0
30	16.5	9	8			
25	12	5.9	5			
20	11	5.3	5			
15	6.2	3.2	1			
10	1.7	0.4	1			

Fall 2011			
	VK826 to VK906		
# particles	2500	1000	100
No PLD	100	86.5	90
30	1.8	3.9	6
25	1.3	4.5	2
20	0.3	3.9	1
15	0	1.1	2
10	0	0.5	0

Winter 2012												
	VK826 to VK906			VK826 to MC885			GB299 to GC140			GB299 to GC234		
# particles	2500	1000	100	2500		100	2500	1000	100	2500	1000	100
No PLD	63	42.6	28	0.4			36.4	34.7	34	20.7	17.3	30
30	13	8.4	5				0.1					
25	9.5	5.4	4				0.1					
20	9	3.8	5									
15	7	2.6	3									
10	2	0.9	1									
